# Phytochemical Synthesis of Silver Nanoparticles by Two Techniques *Using Saturaja* rechengri Jamzad Extract: Identifying and Comparing *in Vitro* Anti-Proliferative Activities

**DOI:** 10.15171/apb.2018.028

**Published:** 2018-06-19

**Authors:** Fataneh Narchin, Kambiz Larijani, Abdolhossein Rustaiyan, Samad Nejad Ebrahimi, Farzaneh Tafvizi

**Affiliations:** ^1^Department of Chemistry, Science and Research Branch, Islamic Azad University, Tehran, Iran.; ^2^Department of Phytochemistry, Medicinal Plants and Drugs Research Institute, Shahid Beheshti University, Evin Tehran, Iran.; ^3^Department of Biology, Parand Branch, Islamic Azad University, Parand, Iran.

**Keywords:** Satureja, Nanoparticle, Green chemistry, Silver, Phytochemical, Cytotoxicity

## Abstract

***Purpose:*** A lot of plants are available which can produce nanoparticles used in medicine, life sciences, and the pharmaceutical industry. The present study aims to introduce safe biological and eco-friendly methods for synthesizing silver nanoparticles (AgNPs) by using Saturaja rechengri Jamzad extract, which can replace traditional chemical methods. In addition, the chemical nature and antimicrobial activities were identified and accordingly the anticancer effects of AgNPs was successfully reported on colon cancer cells (HT-29).

***Methods:*** Light and ultrasound, as two green chemistry techniques were first used for AgNPs synthesis. Then, morphological and crystalline structure of AgNPs was evaluated by scanning electron microscopy (SEM) and X-ray diffraction (XRD) analysis, respectively. In addition, functional groups were determined by using the Fourier transform infrared spectroscopy (FTIR) spectrum. Further, a maximum adsorption of AgNPs was observed in UV-visible spectrum. In the next stage, antibacterial activity of green synthesized AgNPs was evaluated against two pathogenic bacteria including Escherichia coli and Staphylococcus aureus. Finally, the cytotoxicity of AgNPs on HT-29 at different concentrations and times of AgNPs was determined by MTT assay.

***Results:*** The findings indicated that the synthesis of AgNPs by ultrasonic technique leads to smaller particle size and more distribution. Based on the results of MTT test for calculating the IC50%, the anti-proliferative effects of the light and ultrasound AgNPs were observed on HT-29 cell lines depending on the dose and time. Finally, the AgNPs had the most cytotoxicity HT-29 cell lines at 100 μg/ml concentration although the lowest toxicity effect was reported on HEK-293 cell lines at the same conditions.

***Conclusion:*** The change in the concentration, physical and chemical properties of AgNPs including the form and size of particles, and their type of covering and fields can influence the induction of cytotoxicity and morphological change in the treated cells. The present research opens a new horizon on the development of new biological and cytotoxicity agents.

## Introduction

Bio-nanotechnology is regarded as one of the most valuable nanotechnology in the modern era. In addition, it is an interdisciplinary area related to physics, electronic materials, biology, chemistry and medicine, which uses these scientific fields to control matter at the molecular scale.^1^ The use of plan biomass to produce nanoparticles is a relatively simple and cost-effective technique. In addition, the biomass derived from live or dead plants is used as a renewable energy source to generate electricity, biogas, fuel, and the like. Further, the use of biomass for producing nanoparticles is exciting.^2,3^ Generally, this technique helps to protect the environment and reduce the risk of damage for human beings, nature, air and the ecosystem.^4,5^ Nanoparticles used in diagnosing and treating diseases are divided into organic and inorganic category. Organic nanoparticles include albumin, lipid nanoparticle and chitosan, and the like while inorganic nanoparticles are often found as metal nanoparticles including magnetic, copper, gold and silver, etc‏.^6^The use of nanoparticles is important since they are comparable with cellular components in human cells in term of size. It seems that the nature has used materials at a nano-scale to construct biological systems. If someone is supposed to accompany the nature in treating the diseases, he should use materials at a scale which is similar to the nature in some areas like correcting defective genes,^7^ preventing the proliferation of viral genomes^8^ and cancer cell death,^9^ and restoring to cellular metabolism.^10^ Among different synthesized nanoparticles, AgNPs are considered as one of the most widely used drug-delivery nanotechnologies for treating diseases. In this regard, a large body of research was conducted on the related anticancer,^11^ antibacterial,^12^ antifungal,^13^ and antiviral effects.^14^ The results indicated that AgNPs can damage DNA by destructing the mitochondrial respiratory chain in cancer cells, leading to the cell death.^15,16^ However, silver, in itself, can play a minor role or lacks the properties. Compared with macro silver particles, this dual effect of nanoparticles is caused by increasing the areas, along with the reactivity of matter and its compliance with quantum physics and chemistry in nano mode.^17^ Based on green chemical compounds, producing AgNPs by using green chemistry includes choosing the solvent environment, a safe operating environment revivalist, and non-toxic materials for the stability of the nanoparticles (Eshleman, 2011). Hera et al. reported the formation of Ag^+^ from alfalfa biomass in an aqueous solution in a pH-dependent manner.^18^ Mata et al. examined the cytotoxic effects of AgNPs synthesized by the aqueous extract of Abution indicum leaf in the cancer cell line (COLO 205) (human colon cancer) and MDCK (normal) cell *in vitro* conditions and observed that concentration-depend AgNPs, inhibit the growth of this cancer cell line. In addition, it was observed that AgNPs cause morphological changes in chromatin densities and the membranes of treated cells, which ultimately lead to the cell death.^19^ Savory with the scientific name (Satureja hortensis) belongs to the Lamiaceae family. Lamiaceae is one the largest plant families with a global distribution. This genus has 15 species in Iran, among which 9 species such as *S. edmondi, S. sahendica, S. kallarica, S. bachtiarica*,* S. intermedia, S. isophylla, S.atropatana, S. khuzestanica. S. Rechingeri* are exclusive to Iran. Some studies reported carvacrol and flavonoids as the main components of Satureja species^20,21^ including antioxidant properties.^22^ During recent years, a number of studies reported anti-viral properties,^23^ analgesic and anti-inflammatory,^24^ antibacterial and antifungal,^25-27^ antispasmodic and anti-diarrhea,^28^ vasodilatory properties^29^ for different species of this plant, among which we can refer to Satureja rechingeri as one of the endemic species of this plant*.* In this study, the green synthesized AgNPs extracted from *S.rechengeri* were used to determine the antimicrobial and anti-cancer effects on colon cancer cell lines. However, no study has yet addressed different AgNPs synthesis methods of *S.rechingeri* by using light and ultrasonic techniques. Based on the review of the literature, this is the first study addressing the quick and simple synthesis of AgNPs by using *S.rechingeri* extract as a reducing and stable agent to determine its effect on colon cancer cells. It seems that the introduction of drugs used in traditional medicine, especially medicinal herbs, represents a good starting point for the development of research projects to produce new drugs for the treatment of cancer.^30^

## Materials and Methods

### Plant Extract Preparation

The aerial parts of the plant‏ were collected at the flowering stage from a farm owned by Khoraman Company in Lorestan Province (Iran) in July 2016 with the Herbarium code MPH-1348. The fresh plants of *S.rechengri* were dried in the shade for 10 days and then were powdered. In order to prepare aqueous solution of the plant extract, 20 g of plant powder was weighed and was kept for 24 hours in the laboratory conditions after being ionized into 80 ml water. In the next stage, the solution was placed in water bath with 80^o^C for 45 min and was filtered by Watman Filter Paper No. 2. Accordingly, the extract was treated by vacuum rotary evaporation (Rv10 digital, Germany) and was kept at 4°C for use and identification by various techniques in order to remove the extra solvent and obtain a good concentration.

### AgNPs Synthesis 

In order to synthesize AgNPs, the two following methods as green chemistry were used. The solution of silver nitrate salt (AgNO_3_) (Merck, Germany) 0.001 M was prepared in both methods.

- Light: Silver nitrate solution and the extract were mixed with a ratio of 1:4 for the synthesis of AgNPs. The sample was exposed to direct sunlight for 5 min at pH=7. The rapid color change from pale yellow to reddish-brown indicated a reduction of Ag^+^ to Ag^0^ and the formation of AgNPs. Then, the sample was placed at room temperature for 24 hours.
- Ultrasound: One part of AgNO_3_ solution 0.001M was mixed with four parts of the extract (a mix ratio of 1:4) and the same was exposed at ultrasound irradiation of 40 Hz in the dark for three times, each time for 30 min, at 40^o^C and pH= 7 Changes in color from pale yellow to reddish-brown indicated the formation of AgNPs.

### Charactization of AgNPs

#### Spectroscopy (UV-visible) to identify AgNPs

The color change was observed under light and ultrasound conditions, which was very fast and clear in light conditions. The absorption spectra were measured by using a spectrophotometer (JASCO V-670 Spectrophotometer) in the range of 300-600 as silver absorption occurs in this range.

#### XRD diffraction analysis 

X-ray diffraction analysis XRD was used to identify the phase and determine the crystalline structure of AgNPs in the range of ‏ 5> 2Ǿ> 90. Sharp peaks indicated the crystallization properties of the produced sample. The precipitation from the interaction of the aqueous extract of the plant and silver ion was centrifuged three times at 1200 rpm and washed with deionized water. Then, it was dried at 45°C for 24 hours and accordingly used for its identification.

#### Infrared Fourier transform spectrum analysis (FTIR)

In the next stage, the functional groups of AgNPs were determined by ultrasound and light techniques by using Fourier transform spectrometer. This technique involves the identification of functional groups AgNPs in the range of 500-4000 cm^-1^.

#### Scanning electron microscopy 

Scanning electron microscopy SEM is a considered as a technique used for direct observation of AgNPs and estimating their size and surface morphology. This technique is appropriate for identifying the particles with smaller particle size and is regarded as a good standard for determining the validity of other techniques. Further, it provides some information about the structure of particles through electron distribution and dimensions of synthesized nanoparticles. In the next procedure, SEM image of AgNPs was photographed to determine the shape and size of the nanoparticles. To this end, a thin layer of sediment was placed on a gold cloth at a low voltage and under vacuum pressure (5-8 Torr) by using Philips XL30 electron microscope. It was found that the particles are mainly spherical in shape and their diameter is in the range of 44.2 and 65.3 nm, which are stacked together in some areas.

#### Antimicrobial test

#### MIC and MBC evaluation

The Minimum inhibitory concentration (MIC) of synthesized AgNPs light and ultrasound for *Escherichia coli* and *Staphylococcus aureus* was determined by the Mueller-Hinton broth (Merck, Germany) micro dilution method (CLSI, 2010) on 96-well plates with incubation at 37°C for 24 hours. Further, MIC and higher concentrations were cultured on nutrient agar (Merck, Germany) plates and incubated at 37°C for 24 hours in order to evaluate the minimum bactericidal concentration (MBC) of AgNPs light and ultrasound. Then, the MIC value was determined for the least dose of synthesized AgNPs, which prevented the growth of tested bacteria. In order to achieve a better result, the test was repeated twice.

#### Cytotoxicity effect

#### Preparation of cell lines and culture 

The HT-29 cells (Human colorectal adenocarcinoma, IBRC C10097) and Normal HEK-293 (IBRC C10139) were purchased from National Cell Bank in Iranian Biological Resource Center (Cell Bank of Pasteur Institute Tehran-Iran). Then, they were defrozen and cultured in the IMPR environment enriched with 10% SBF and bovine serum and 1% penicillin –streptomycin. After the cell lines were grown sufficiently, they were counted and a total number of 10^4^ cell lines were cultured in a 96-well plate and incubated within incubators (Binder) at 37°C and 5% CO_2_.

#### MTT Assay

MTT assay is a colorimetric method based on the revival and break of yellow crystals with chemical formula 3- [4, 5-dimethylthiazol-2-yl] -2, 5-diphenyl-] which are regenerated by tetrazolium bromide enzyme succinate dehydrogenase in the active cellular mitochondria and the formation of insoluble blue crystals. The color is proportional to the cellular activity and the number of living cells.^31^ After 24 hours, 100 ml of the cultured cells was covered with 100 ml of AgNPs of salt with a concentration of 0.5 mg ml for 4 hours by using a foil.‏ Then, the cell cytotoxicity was estimated through colorimetric method (MTT) on colon cancer cells HT-29 and healthy cells HEK-293 by measuring the light and ultrasound absorbance based on the concentration of AgNPs. In the next stage, the results were compared with the rate of cell survival. In addition, the cells were cultured in a 96-well plate with a density of 10^4^cells per plant and were incubated in a CO_2_ incubator (37^o^C) for 24 h. Further, HT-29 cancer cells were treated with different concentration of AgNPs synthesized from the aqueous extract of the plant for 24 and 48 h, respectively. After treating the samples, 10 μl of MTT solution (5 mg/ml) was added to each plate containing AgNPs-treated cells and incubated at 37°C and 5% CO_2_ for 5 h. Finally, DMSO (0.5 μl ) was added to each well until dissolving the purple crystals, and the light absorbance was read by a spectrophotometer at 570 nm after 20 min at room temperature.^32^ In addition, the inhibitory concentration (IC_50%_) for the samples was determined as follows:

$$Percentage\;of\;cell\;viability\;\%=\frac{\text{Average absorbance by the treatment sample}}{\text{Average absorbance by the control sample}}\times 100$$

#### Statistical analysis

The quantitative data was evaluated using SPSS software (Version 16) (SPSS Inc., Chicago, IL) and the results were analyzed by ANOVA at a software using statistical tests at significant level. The data were determined as mean ± SD and p < 0.05 was considered statistically significant.

## Results and Discussion

### Synthesis Charactization of AgNPs

This study was designed to synthesize AgNPs by using *S.rechengri* extract based on light and ultrasonic techniques, which are economical and ecofriendly. In addition, the enormous potentials of the nature can be employed for synthesizing nanoparticles in these two techniques, without damaging the environment. Recently, a lot of plant extracts have been studied for AgNPs synthesis. However, in the present study, aqueous extracts were extracted from the aerial parts of dried *S.rechengri* for AgNPs synthesis and the extracts prepared by using a solution of AgNO_3_ 0.001 M (from Merck Company, Germany) with a purity of 99/99% were mixed. In order to synthesize the AgNPs from the obtained solution, both light and ultrasound techniques (absence of light) were used. The results showed a change in color of the extract from pale yellow to dark brown at room temperature and the formation of a colloidal mix of AgNPs. The observation of the color change due to the interaction of herbal extracts and silver salt solution was similar to the results of the study by‏ Reddy and Gandhi (2012) and is considered as the first microscopic signs of the production of AgNPs.^33^ The speed of the reaction reducing Ag^+^ in the aqueous extract of *S.rechengri* by using the light technique was found to be higher than that of ultrasound technique. Further, the color change in the light technique was considerably clearer than of the ultrasound technique. In the ultrasound techniques, the silver nitrate solution and the extracts were exposed to ultrasonic irradiation with intensity (40 Hz) at 40^o^C for 30 minutes while the solution was exposed to direct sunlight (visible waves) for 5 min in the optical techniques. The completion of the synthesis of AgNPs at higher speeds is regarded as one of the benefits of these methods. Furthermore, UV-visible absorption occurred in the 300-600 nm region for both methods. The observation of absorption in the region of x nm confirms the synthesis of AgNPs. In the present study, the presence of AgNPs peak at a wavelength of 430 nm is consistent with the observations made by Song and Kim (2009) and Sathyavathi et al (2010).^34^ Todays, there are many plants which are used for synthesizing metal nanoparticles such as Ag.^35,36^ However, only one of these species, *S.rechengri* is used for AgNPs synthesis although Satureja has many species. Biological organisms are significant at both capping and oxidant agents in the synthesis of AgNPs. A large body of research indicates that the synthesis of AgNPs by using bioorganic compounds is successful. In addition, the reduction of Ag^+^ by biomolecules including the cell extract such as enzymes, proteins, vitamins, amino acids, and polysaccharides are compatible with the nature.

Dehghanizade et al. (2017) investigated the synthesis of AgNPs using Anthemis atropatana extract and its antimicrobial and anticancer effects, and showed that biosynthesis of AgNPs by green chemistry is easily affordable and there is no require any reducing agents.^37^ Extensive studies have shown that a combination of phenolic acids and amine acids, flavonoids, enzymes and proteins present in the plant extract may be the main cause for rapid and easy reduction of metallic silver ions,^38^ which play an important role in the synthesis of AgNPs.^39,40^ However, the chemical synthesis of such nanoparticles brings about many environmental disadvantages and problems, which is not environment-friendly.^41^

### UV-Visible

**Figure 1:**
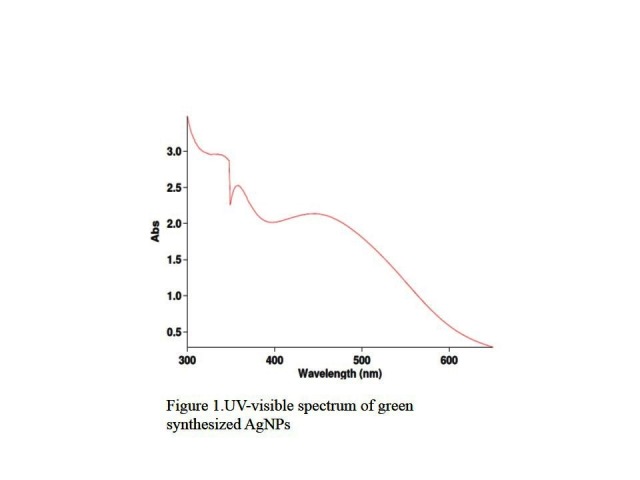
UV-visible spectrum of green synthesized AgNPs

The UV-visible spectrum was used to determine the absorption wavelength of AgNPs, which indicated a high absorption occurred in the wavelength 430 nm. Based on the theory of the effect of quantum restraint, lower wavelength leads to higher energy of the wave and smaller particle size.^42^ Thus, this spectrum confirms the formation and fineness of AgNPs (Figure 1).

### XRD analysis of AgNPs

**Figure 2:**
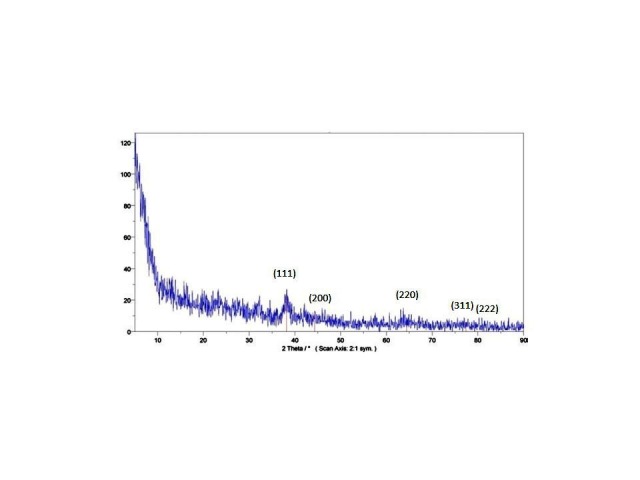
XRD pattern of AgNPs synthesized. Schema of XRD peak related to face-centered cubic (fcc) (111), (200), (220), (311) and (222) in sample silver spectrum.2θ angles related to silver schema are observed in 38.01, 44.5, 64.78, 77.7 and 82.1.

In addition, XRD diffraction was used to determine the crystalline structure of the synthesized AgNPs. The relative intensity of each peak represents the type of synthesized AgNPs and their phase qualitatively. As observed in Figure 2, the peak intensity in regions 111, (200), (220), (311), and (222) confirms the regular crystal structure of the synthesized AgNPs at angles 5> 2 Ǿ > 90. Further, sharp peaks represent the crystalized nature of the produced samples. Furthermore, the analysis of the diffraction patterns of the irradiation could confirm the cubic crystal structure of the samples.^43^

### FTIR spectroscopy

**Figure 3:**
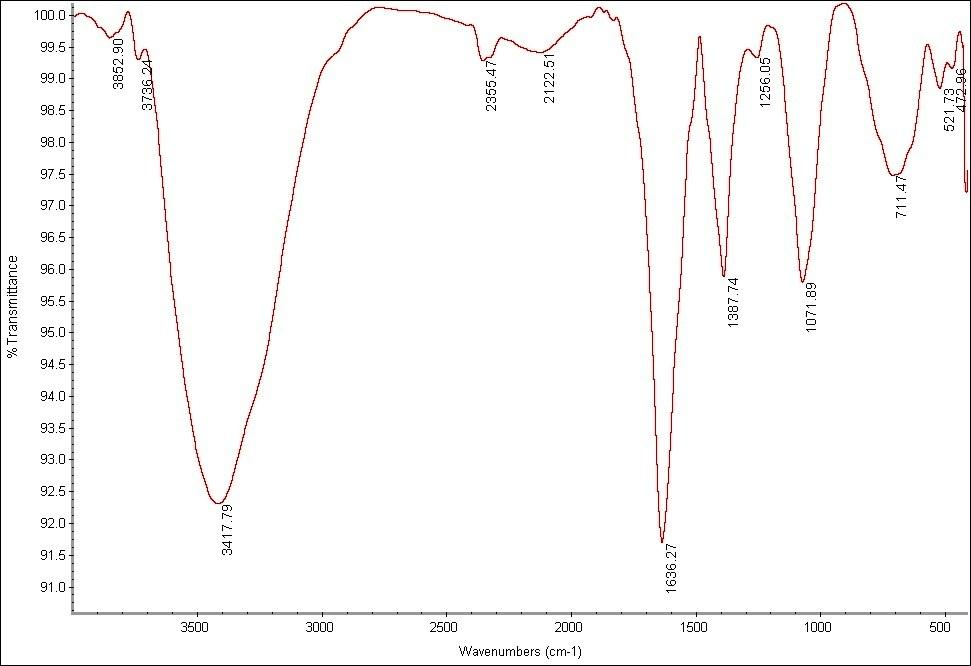
FTIR of AgNPs. The analysis of the AgNPs showed the stretching frequencies in 472.99, 521.73, 711.74, 1071.89, 1387.74,16, 36.27 and 3417.79 cm^-1^.

In order to study the tensile and flexural frequency of the bonds in the synthesized AgNPs, the Fourier transform infrared (FTIR) spectrometer was used (Figure 3). In general, the peaks of 3417.79 and 1636.27 cm-represent tensile and flexural modes of the absorbed surface water (H-O-H) of this blend, respectively. Additionally, the bending vibration mode of Ag-O in the peak numbers of 521.73 and 472.96 cm-confirms the formation of AgNPs. In this spectrum, the peak close to the wave number 600 cm- is related to two split peaks. It seems that this splitting is the result of separating energy levels of AgNPs, which are quantized.

### SEM and EDX analysis

**Figure 4:**
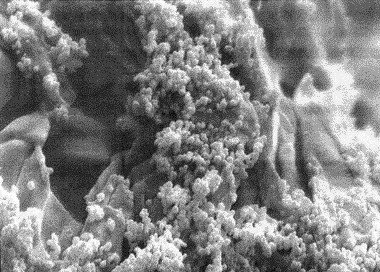
SEM analysis of AgNPs using green chemistry in Ultrasound technique.

**Figure 5:**
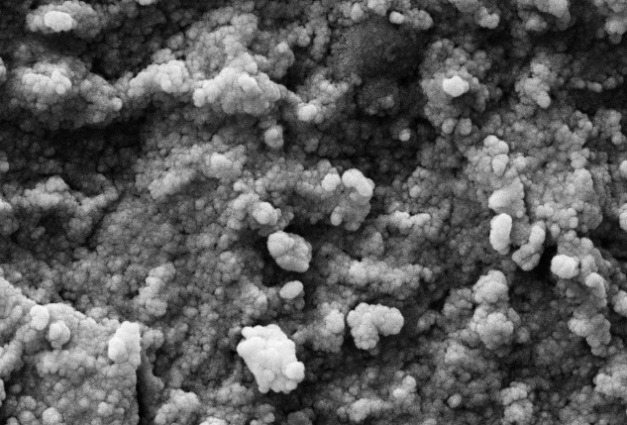
SEM analysis of AgNPs using green chemistry in light technique.

**Figure 6:**
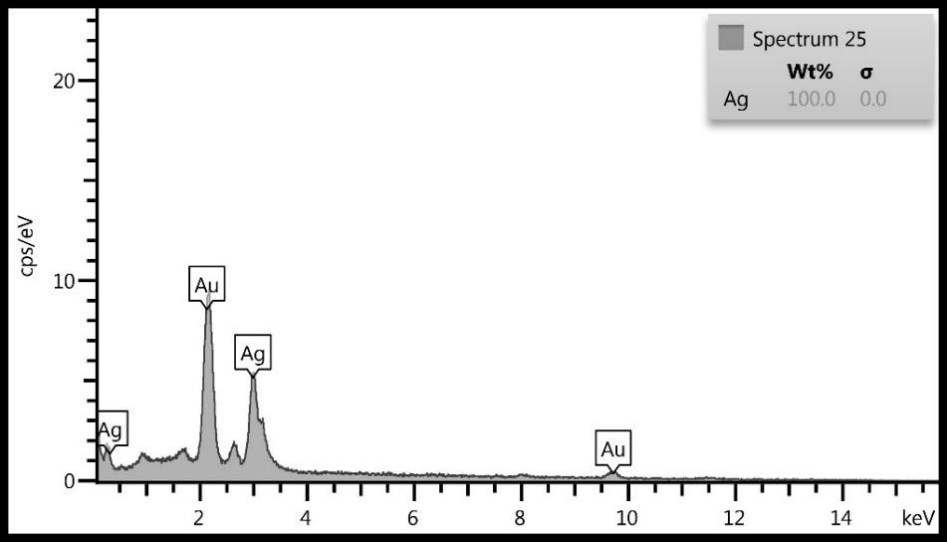
EDS analysis of AgNPs green chemistry

The analysis of the structure and morphology of nanoparticles, as well as their size and surface morphology, was performed by SEM microscopy. The synthesized AgNPs in the colloidal solution were exposed to irradiation by using light and ultrasound techniques. The comparison of the produced spherical AgNPs indicated that the synthesized AgNPs produced through ultrasonic irradiation technique are smaller in size and more spherical in shape and have a better distribution. The particle size in the ultrasound technique ranges from 44 to 65.3 nm and is 54.75 nm on average (Figure 4) while the particle size in the light technique ranges from 57.1 to 88.5 nm, with an average of 72.8 nm (Figure 5). Further, the Energy Dispersive X- ray Spectroscopy (EDS) spectrum was determined to indicate that it is coated with 100% silver (Figure 6).

### Antibacterial test

The antibacterial activity of the synthesized AgNPs from the aqueous extract of *S.rechengri* was considered based on two bacterial pathogens including* Escherichia coli* and *Staphylococcus aureus*. The results were evaluated for the lowest inhibitory concentration (MIC) of AgNPs in terms of the growth of selected bacteria. The results of some studies indicated that the synthesized AgNPs cause bacterial cell death by destructing the cell membrane of the bacteria such as Staphylococcus and E-coli at a given concentration (Table 1) while bacterial growth are inhibited in other concentrations.^44,45^Another proposed mechanism for the death of bacteria is related to the AgNPs release of Ag^+^which can bind biological macromolecules including oxygen, nitrogen, and sulfur, and disrupt the function of biological molecules, leading to the death of the bacterial cell.^46^

### MTT assay

**Figure 7:**
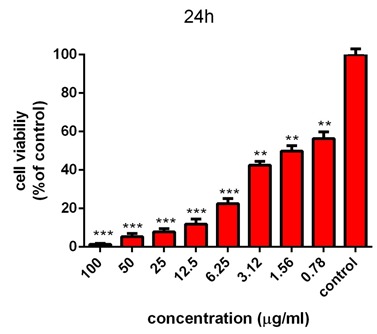
Survival percentage of HT-29 cells against different concentrations of light AgNPs within 24 hours. Results have been reported as survival rate compared with control samples (n=3 P <0.001 ***, P < 0.01**, P <0.05 *).

**Figure 8:**
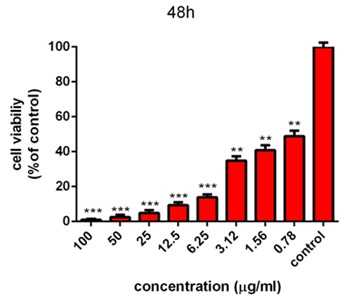
Survival percentage of HT-29 cells against different concentrations of light AgNPs within 48 hours. Results have been reported as survival rate compared with control samples. (n=3 P <0.001 ***, P < 0.01**, P <0.05 *).

**Figure 9:**
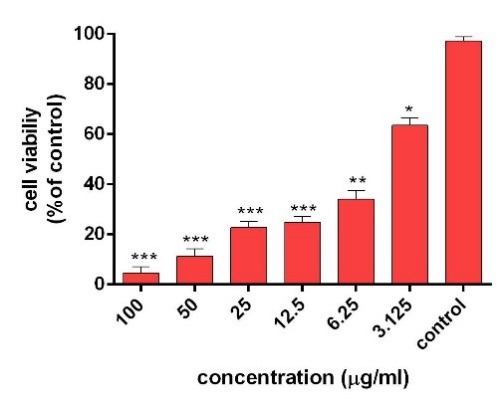
Survival percentage of HT-29 cells against different concentrations of ultrasound AgNPs within 24 hours. Results have been reported as survival rate compared with control samples (n=3 P <0.001 ***, P < 0.01**, P <0.05 *).

**Figure 10:**
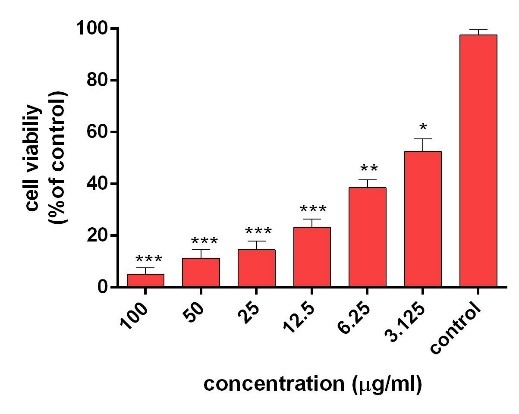
Survival percentage of HT-29 cells against different concentrations of ultrasound AgNPs within 48 hours. Results have been reported as survival rate compared with control samples. (n=3 P <0.001 ***, P < 0.01**, P <0.05 *).

The cytotoxicity of AgNPs was first estimated with colorimetric method (MTT) by measuring the optical density based on the concentrations (3.125-6.5-12.5-25-50-100 μg/ml) of green synthesized AgNPs through light and ultrasound techniques. Then, the results were compared with cell survival rate. In addition, the effects of synthesized AgNPs on colon cancer cell lines (HT-29) and normal cells (HEK-293) were evaluated. In the next procedure, the survival rate of the treated cells with AgNPs was measured, compared to the survival rate of the untreated (control) cells during 24 and 48 hours after the treatment. Based on the results, the reduction of the cell survival rate was observed with increasing concentrations of AgNPs. In addition, at the highest dose (100 µg /ml), the cell viability for the cells treated by synthesized AgNPs by light and ultrasound techniques were found to be at the lowest rate. Tables 2 and 3 represent the results of biological power of the cells in terms of the color change in salt dimethylthiazol by light and ultrasound AgNPs, respectively. Further, the values were obtained on the mean ± SD and the mean differences were compared at the significance level of P ≤ 0.05 (ANOVA and t-test). Figures 7, 8, 9 and 10 illustrate the dose dependence of anticancer effects of AgNPs on HT-29 cell lines.

**Table 1:** Minimum inhibitory concentration (MIC μg /ml) against the two bacteria withsynthesized AgNPs by light and ultrasound

**Bacteria**	**Minimum inhibitory concentration (µg/ml)**	
	**light**	**ultrasound**
**Escherichia coli**	6.25	-
**Staphylococcus aureus**	1.56	100

**Table 2:** The cell viability for HT-29 cells with light AgNPs during 24 and 48 hours by MTT.

**Concentrations (µg/ml)**	**3.125**	**6.25**	**12.5**	**25**	**50**	**100**
**Cell viability (% of control)**	^a^42.5‏ ±‏ 0.36	22.5‏ ±‏ 0.49	11.75±0.44	7.7±0.34	5.25±0.27	1.27±0.12
	^b^34.25±‏ 0.44	13.7‏ ±‏ 0.42	9.2‏ ±‏ 0.37	4.7±0.28	2.5±0.22	1±0.09

* Percentage of cell survival within 24 (a) and 48 (b) hours

**Table 3:** The cell viability for HT-29 cells with ultrasound AgNPs during 24 and 48 hours by MTT.

**Concentrations (µg/ml)**	**3.125**	**6.25**	**12.5**	**25**	**50**	**100**
**Cell viability (% of control)**	^a^63.5±‏ 0.38	37.5‏ ±‏ 0.88	24.75±0.78	22.25±0.28	11.5±0.58	4.5±0.36
	^b^50.2±‏ 0.57	36.6±‏ 0.52	23.8‏ ±‏ 0.44	14.7±0.25	10.3±0.15	3.9±0.02

* Percentage of cell survival within 24 (a) and 48 (b) hours

**Figure 11:**
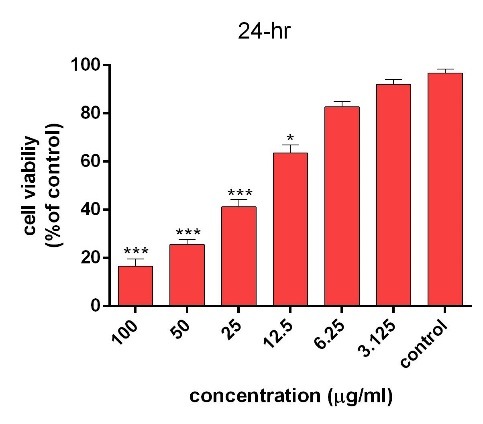
Survival percentage of normal HEK-293 cells against different concentrations of AgNPs within 24 hours. Results have been reported as survival percentages compared with control samples (n=3 P <0.001 ***, P < 0.01**, P <0.05 *).

The induction of cytotoxicity of ultrasound AgNPs was observed at higher concentrations and was found to be more intense than that caused by light AgNPs. The results indicated that the effect on cell lines HT-29 relies on their concentration, in addition to the size and shape of AgNPs, Further, the results of calculating the IC_50%_ for the light AgNPs showed that such nanoparticles can inhibit 50% of the cells during 24 and 48 hours at concentrations of 1.3 and 0.71 µg /ml. Furthermore, light AgNPs within 48 hours with lower doses can prevent the growth of 50% of the cancer cells HT-29, indicating a significant difference during 24 hours. Additionally, the results of ultrasound AgNPs in 24 and 48 hours at the concentrations of 4.7 and 2.6 µg /ml indicated that these AgNPs can inhibit 50% of the cells. In addition, the MTT results of synthesized AgNPs through light and ultrasound technique were performed on a healthy HEK-293 cell line. The values were determined for ultrasound AgNPs 3.125-6.5-12.5-25-50-100 μg/ml for 24 hours (Table 4). Based on the results, both AgNPs had more effective cytotoxicity against HT-29 cells than HEK-293 cell lines. Figure 11 displays the concentration dependence of cytotoxicity effect of AgNPs on HEK-293 cell line.

**Table 4:** The cell viability for HEK-293 cells with AgNPs during 24 hours by MTT.

**Concentrations (µg/ml)**	**3.125**	**6.25**	**12.5**	**25**	**50**	**100**
**cell viability (% of control)**	^a^94.76±‏ 0.43	87.97±0.25	63.56±0.34	25.88±0.25	22.21±0.28	17.76±0.21

* Percentage of cell survival within 24 (a) hours

## Conclusion

The present study seek to address the use of nano-synthesis compounds of *S.rechengri* for the preparation of AgNPs. In addition, its chemical properties and effectiveness for treating colon cancer cell lines were taken into consideration. The results indicated that targeted synthesized AgNPs include desired properties, shape, size and useful biological effects. Furthermore, various methods of synthesizing AgNPs by considering biocompatibility as a priority were examined in this study. Therefore, *S.rechengri*‏ has been first used as a living factory for the production of AgNPs. The importance of these techniques is related to their simplicity and speed of AgNPs synthesis, irrespective of the need for using hazardous chemicals. In addition to medicinal properties, *S.rechengri*‏ has a unique feature anticancer, which play a significant role in destructing the cancer cell line HT-29. Finally, it can be used to produce AgNPs for medical and pharmaceutical purposes. It suggested that further researches were performed for AgNPs pharmaceutical effects.

## Acknowledgments

We would like to appreciate all colleagues, in particular Dr. Hassan Noorbarzgan and Dr. Rahem Khoshbakht who helped us in analyzing biological data (MIC & MTT) and Dr. Amir Mirzaie for useful guidance. This research was sponsored by Islamic Azad University, Science and Research Branch of Tehran, Iran.

## Ethical Issues

This project was conducted in accordance with the principles laid down in the Helsinki Protocol and approved by the Ethics Committee of the Council of Science and Research of the Islamic Azad University.

## Conflict of Interest

The authors report no conflicts of interest in this study.
